# *β-Sitosterol* attenuates obstructive sleep apnea-related myocardial injury via MALAT1-mediated HIF-1α regulation

**DOI:** 10.3389/fphar.2025.1692758

**Published:** 2025-10-15

**Authors:** Runhua Wu, Naijie Chen, Jingyi Li, Dan Liu, Feng Wang, Qin Chen

**Affiliations:** ^1^ College of Integrated Medicine, Fujian University of Traditional Chinese Medicine, Fuzhou, Fujian, China; ^2^ Clinical Skills Teaching Center, Fujian University of Traditional Chinese Medicine, Fuzhou, Fujian, China

**Keywords:** β-sitosterol, obstructive sleep apnea, myocardial injury, MALAT1, HIF-1α, chronic intermittent hypoxia

## Abstract

**Background:**

Obstructive sleep apnea (OSA) is associated with chronic intermittent hypoxia (CIH), which contributes to myocardial injury. Although continuous positive airway pressure (CPAP) is the standard therapy, patient compliance remains challenging. *β-Sitosterol*, a natural phytosterol with anti-apoptotic properties, has shown potential cardioprotective effects, but its role in OSA-related myocardial injury remains unclear.

**Methods:**

A CIH rat model and hypoxic H9c2 cardiomyocytes were used to evaluate the effects of *β-sitosterol*. Myocardial injury was assessed via heart-to-body weight ratio, histopathology (H&E, Masson, TUNEL staining), and apoptosis markers. Molecular mechanisms involving lncRNA MALAT1 and HIF-1α were investigated using RT-qPCR, Western blot, RNA-FISH, luciferase assays, and gain-of-function experiments.

**Results:**

*β-Sitosterol* treatment significantly reduced myocardial fibrosis, apoptosis, and structural damage in CIH rats in a dose-dependent manner. *In vitro*, it enhanced cell viability and suppressed apoptosis under hypoxic conditions. Mechanistically, *β-sitosterol* downregulated MALAT1 and HIF-1α expression. Although MALAT1 and HIF1A co-localized in the cytoplasm, no direct binding was detected. Overexpression of MALAT1 abolished the protective effects of *β-sitosterol* and reactivated HIF-1α/Bax/caspase-3 signaling.

**Conclusion:**

*β-Sitosterol* attenuates CIH-induced myocardial injury by inhibiting the MALAT1/HIF-1α axis, suggesting its potential as a therapeutic agent for OSA patients with limited tolerance to conventional therapies.

## 1 Introduction

Obstructive sleep apnea (OSA) is a prevalent sleep-disordered breathing condition characterized by recurrent upper airway obstruction during sleep, resulting in apnea or hypopnea, which leads to chronic intermittent hypoxia (CIH) and cardiac damage (Arnaud et al., 2020). The main clinical manifestations of OSA include snoring at night, daytime sleepiness, dry mouth in the morning, and headache, all of which have a great impact on the work and life of patients (Sunitha and Aravindkumar, 2009). The prevalence of OSA in the general population ranges from 13% to 33% in males and 6%–19% in females, and mostly occurs in middle-aged and elderly individuals (Senaratna et al., 2017; Fietze et al., 2019). Continuous positive airway pressure (CPAP) is considered the standard treatment for OSA (Sun et al., 2014). It offers positive pressure ventilation to the airway via a mask in order to re-expand the initially collapsed airway and subsequently alleviate the apnea caused by the collapse of the airway. However, certain patients encounter difficulties adhering to CPAP treatment due to issues with the mask (Weingarten, 2022). Consequently, there is an urgent necessity to identify alternative treatments for OSA.

Our preliminary studies have confirmed that Yi Qi Hua Tan Qu Yu formula effectively improves the clinical symptoms of OSA patients. However, the underlying mechanism remains unclear (Chen et al., 2016). *β-Sitosterol* (C_29_H_50_O) is the primary active ingredients of the multi-flavored herbs in the prescriptions, including *Carthamus tinctorius L., Codonopsis pilosula (Franch.) Nannf, Angelica sinensis (Oliv.) Diels,* and *Ligusticum chuanxiong hort*. *β-Sitosterol* exhibits a range of physiological activities, such as antibacterial, antioxidant, anti-inflammatory, anti-depressant and anti-tumor effects (Khan et al., 2022; Nirmal et al., 2012). A study conducted by Rajavel et al., has demonstrated that *β-sitosterol* induces ROS accumulation and mitochondria-mediated apoptosis in cancer cells by inhibiting Trx/Trx1 reductase, which alleviates the progression of non-small cell lung cancer cells (Rajavel et al., 2018). Furthermore, *β-sitosterol* has been found to have a protective effect on the occurrence of N-diethylnitrosamine combined with ferric nitrilotriacetate induced renal cancer by accelerating apoptosis of renal cancer cells (Sharmila and Sindhu, 2017). Additionally, *β-Sitosterol*, a major bioactive component in herbs like *C. tinctorius* and *C. pilosula* used for cardiovascular protection, was selected due to its reported anti-apoptotic effects in myocardium (Lin et al., 2020; Wong et al., 2014). However, the specific function and mechanism of *β-sitosterol* in CIH-induced myocardial injury remains unclear.

Long non-coding RNAs (lncRNAs) are a class of transcripts longer than 200 nucleotides that regulate gene expression at epigenetic, transcriptional and post-transcriptional levels (Sun et al., 2018). LncRNAs are involved in the progression of various diseases. A recent investigation has confirmed that lncRNA MALAT1 promotes myocardial cell apoptosis by regulating miR-145/Bnip3 axis, thereby contributing to ischemia/reperfusion myocardial injury (Zhao et al., 2017). Specifically, MALAT1 acts as an endogenous sponge to adsorb miR-200a-3p and downregulate the level of miR-200a-3p, thus promoting the inflammatory damage of cardiomyocytes during ischemia/reperfusion injury (Sun and Zhang, 2019).

The pathophysiological changes associated with chronic intermittent hypoxia/reoxygenation resemble the process of ischemia/reperfusion injury. The role of MALAT1 in CIH still remains exploration. Additionally, hypoxia-inducible factor-1α (HIF-1α) is a crucial regulator of hypoxia response in mammalian cells. Liu et al. have revealed that the levels of apoptosis-related proteins Bnip3, Bax, and caspase-3 are elevated in hypoxia-treated H9c2 cells, which induces cardiomyocyte apoptosis (Liu et al., 2016). The apoptosis of cardiomyocytes accelerates the occurrence and development of cardiovascular diseases such as heart failure and ischemic heart disease (Meng et al., 2019). Using starBase database, we predicted the relationship between MALAT1 and HIF-1α, and found that there were binding sites between MALAT1 and HIF-1α. Thus, we sought to test whether *β-Sitosterol* affected CIH-induced myocardial injury by regulating MALAT1 and HIF-1α expression.

## 2 Materials and methods

### 2.1 Animal protocols

Sprague Dawley (SD) male rats, weighing 180 ± 20g, were purchased from Hangzhou Medical College (Hangzhou, China). The present research was approved by the Ethics Committee of Fujian University of Traditional Chinese Medicine. Rats were subjected to intermittent hypoxia treatment in an animal intermittent hypoxia incubator (Attendor-140 Pro, Ningbo, China) to construct CIH rat model (Wu et al., 2024). Each experimental group consisted of n = 8 rats. The concentration of oxygen in the incubator was sustained from 5.1% to 21%. The hypoxia/reoxygenation cycle (120 s) was contained 4 periods: (1) oxygen concentration decline period (50 s); (2) hypoxic maintenance period (20 s); (3) oxygen concentration rise period (30 s); (4) normoxic maintenance period (20 s). Rats were subjected to hypoxia/reoxygenation cycle for 30 times/hour, and 8 h/day for 8 weeks. At the same time of hypoxia/reoxygenation treatment, rats were administered intragastrically with low, middle and high dose (10, 15, 20 mg/kg) of *β-Sitosterol* (dissolved in olive oil) (MedChem Express, Monmouth Junction, NJ, United States of America) for 8 weeks. Doses of *β-sitosterol* were selected based on preliminary experiments and previous studies showing efficacy in cardiovascular models (Lin et al., 2020; Wong et al., 2014). The uneven spacing reflects the nonlinear dose-response relationship observed in pilot studies. CIH rats were received high dose (20 mg/kg) of *β-Sitosterol* treatment, and injected with lentivirus (LV)-mediated MALAT1 overexpression vector (LV-MALAT1) or LV-mediated empty vector (LV-NC) into caudal vein. The normal control group received intermittent normoxia and an equivalent volume of olive oil. After 8 weeks of modeling, rats were euthanized with injection of pentobarbital sodium. Myocardial tissues were separated from rats and weighed. The ratio of myocardial tissues weight/body weight was calculated. Subsequently, myocardial tissues were subjected to fixation and embedding for histochemical analysis, or stored at −80 °C for quantitative real-time PCR (qRT-PCR) and Western blotting.

### 2.2 Histochemical analysis

Paraffin embedded myocardial tissues were sectioned, and then subjected to dewaxing and hydration. Myocardial tissue sections were stained with Hematoxylin and Eosin Staining Kit (Beyotime, Shanghai, China), Masson Staining Kit (SenBeiJia, Nanjing, China) and Colorimetric TUNEL Apoptosis Assay Kit (Beyotime) following the manufacturer’s instructions. The pathological changes, fibrosis and apoptosis of myocardial tissues were evaluated.

### 2.3 Cell culture

H9c2 cells (#CRL-1446; ATCC, Manassas, VA, United States of America) were cultured in DMEM (GIBCO, Grand Island, NY, United States of America) containing 10% fetal bovine serum (GIBCO) and 1% penicillin/streptomycin (Sangon, Shanghai, China) at 37 °C and 5%CO_2._ Cells were exposed to CIH environment (repeated cycles of 5% O_2_ for 5 min; followed by 21% O_2_ for 5 min) (Smartor-118pro, Ningbo, China), and lasting for 24 h. CIH-treated cells were allowed to incubate with 1, 3 and 10 μM of *β-Sitosterol*. *In vitro* doses were chosen based on prior reports of *β-sitosterol’s* anti-apoptotic effects in cardiomyocytes (Wong et al., 2014) and our own viability assays. Cells were pre-treated with *β-sitosterol* or vehicle for 1 h before hypoxia exposure. Cells were incubated in normoxia conditions (5%CO_2,_ 95% air gas mixtures) as control.

### 2.4 Cell transfection

The pcDNA3.1 vector containing full length of MALAT1 (pcDNA-MALAT1) was used for MALAT1 overexpression (GenePharma, Shanghai, China). Empty vector served as control. H9c2 cells were transfected with vectors utilizing Lipofectamine 2000 Reagent (Thermo Fisher Scientific, Waltham, MA, United States of America). H9c2 cells were transfected with pcDNA-MALAT1 or vector 24 h prior to hypoxia exposure. *β-Sitosterol* was added at the onset of hypoxia.

### 2.5 RT-qPCR

Using TRIzol reagent and Cytoplasm and Nuclear RNA Purification Kit (Norgen Biotek, St. Catherines, Canada), total, cytoplasm and nuclear RNA were isolated from H9c2 cells and myocardial tissues. Reverse transcription and PCR reactions were conducted utilizing TransScript Two-Step RT-PCR SuperMix (Transgen, Beijing, China). Primer sequence (5′-3′) were listed as follows:

MALAT1:

Forward-AAAGCAAGGTCTCCCCACAAG.

Reverse-GGTCTGTGCTAGATCAAAAGGCA;

HIF1A:

Forward-TCCTATGTGCTGGCTTTGG.

Reverse-AGTGTACCCTAACTAGCCG;

GAPDH:

Forward-GGAGCGAGATCCCTCCAAAAT.

Reverse-GGCTGTTGTCATACTTCTCATGG.

The relative expression of genes was normalized to GAPDH and analyzed by 2^−ΔΔCt^ method.

### 2.6 Western blotting

Applying RIPA buffer (Thermo Fisher Scientific), total proteins were extracted from H9c2 cells and myocardial tissues. Proteins were run on a 10% SDS-PAGE gel, and then transferred to PVDF membranes. The membranes were gradually incubated with primary antibodies at 4 °C overnight and secondary antibody at room temperature for 1 h. The antibodies, anti-HIF-1α (1:1000 dilution; #ab179483; Abcam, Cambridge, MA, United States of America), anti-Bax (1:1000 dilution; #ab182734; Abcam), anti-cleaved caspase-3 (1:1000 dilution; #PA5-114687; Thermo Fisher Scientific), β-actin (1:2000 dilution; #ab8227; Abcam) and goat anti-rabbit IgG (1:5000 dilution; #ab6721; Abcam), were used in Western blotting. β-actin served as loading control. The immunoreactive bands were visualized by enhanced chemiluminescence reagent.

### 2.7 Cell viability and apoptosis

Applying Cell Counting Kit-8 (Beyotime), cells were incubated at 96-well plate for 48 h. Then, cells were treated with CCK-8 reagent at 37 °C for 1 h. The absorbance value of each well at 450 nm was detected by Microplate reader (BioTek, Winooski, VT, United States of America). One Step TUNEL Apoptosis Assay Kit was used to examine cell apoptosis. Cells were incubated with TUNEL reagent at 37 °C in dark for 1 h. Nucleus were stained with 1 μg/mL DAPI for 10 min. The fluorescence of cells was observed applying fluorescence microscope (Olympus, Tokyo, Japan).

### 2.8 Luciferase reporter assay

The psiCHECKTM-2 vector (Promega, Madison, WI, United States of America) carrying the wild type/mutant type (WT/Mut) sequence of HIF1A that targeting MALAT1 was constructed, generating the vectors HIF1A-WT-1, HIF1A-WT-2, HIF1A-Mut-1 and HIF1A-Mut-2 (GenePharma). 293T cells were transfected with luciferase vectors of WT/Mut HIF1A and pcDNA-MALAT1 or Vector. Using a luciferase reporter assay system (Promega), the relative luciferase activity of 293T cells was examined.

### 2.9 RNA fluorescence *in situ* hybridization (FISH)

FISH probes specifically targeting MALAT1 or HIF1A was designed and synthesized by GenePharma. FISH Kit (GenePharma) was employed to examine the localization of MALAT1 and HIF1A. H9c2 cells were fixed with 4% paraformaldehyde, followed by permeabilization with 0.5% Triton X-100. Following 30 min of pre-hybridization, cells were hybridized with 1 µM FITC-labeled MALAT1 and 1 µM Cy3-labeled HIF1A at 37 °C overnight. Nucleus were stained with 1 μg/mL DAPI for 10 min. Finally, the fluorescence of cells was visualized under a fluorescence microscope.

### 2.10 Statistical analysis

Each assay was performed for 3 times. Data were expressed as mean ± SD and analyzed by SPSS 22.0 statistical software (IBM, Armonk, NY, United States of America). Two-tailed Student’s t*-*test and one-way ANOVA were used to analyze the statistical difference. *P* < 0.05 was considered as a significant difference.

## 3 Results

### 3.1 β-Sitosterol alleviated CIH-induced myocardial injury in rats at dose-dependent manner

**FIGURE 1:**
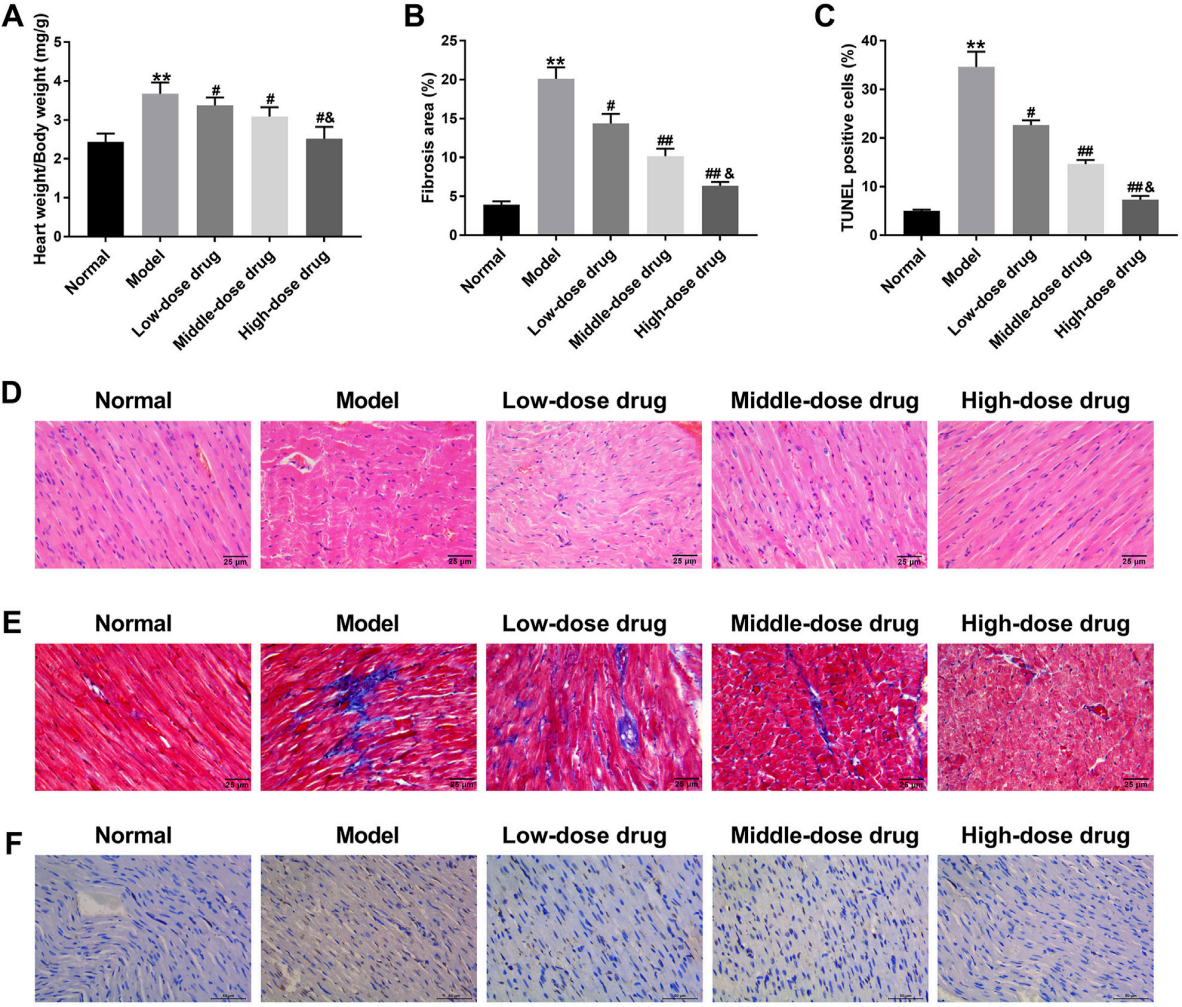
*β-Sitosterol* alleviates CIH-induced myocardial injury in rats CIH rats were administrated with intermittent hypoxia, followed by low, middle and high dose of β-Sitosterol treatment. Rats exposed to normoxia (Normal) received olive oil as vehicle control. **(A)** Ratio of heart weight to body weight (HW/BW) in rats exposed to normoxia (Normal), CIH (Model), or CIH with low/middle/high-dose β-sitosterol treatment (10/15/20 mg/kg). **(B)** Quantitative analysis of fibrosis area in myocardial tissues. **(C)** Quantitative analysis of apoptosis rate (TUNEL staining). **(D)** Representative H&E staining showing myocardial pathology (black arrow: wavy arrangement; yellow arrow: necrotic cells; scale bar: 50 μm). **(E)** Representative Masson’s trichrome staining (blue: collagen fibrosis). **(F)** Representative TUNEL staining (green: apoptotic cells; red: nuclei; scale bar: 20 μm) and **(F)** quantitative analysis of apoptosis rate. Data are presented as mean ± SD; n = 8 per group. **P < 0.01 vs Normal; #P < 0.05, ##P < 0.01 vs Model; ^&^ *P* < 0.01 vs Middle-dose drug.

To explore how *β-Sitosterol* ameliorated CIH-induced myocardial injury, a CIH rat model was constructed, and received different dose of *β-Sitosterol* treatment. The heart weight/body weight was significantly increased in CIH rats. *β-Sitosterol* treatment reduced the heart weight/body weight in CIH rats, especially high dose of *β-Sitosterol* (Figure 1A). Subsequently, we sought to examine if *β-Sitosterol* affected the myocardial fibrosis and apoptosis of CIH rats. Using Masson staining and TUNEL staining, we found that CIH rats exhibited severe myocardial fibrosis and apoptosis, which was effectively inhibited by *β-Sitosterol* at dose-dependent manner. High dose of *β-Sitosterol* significantly repressed the myocardial fibrosis and apoptosis in CIH rats with respect to middle dose of *β-Sitosterol* (Figures 1B,C,E,F). Moreover, the pathological changes of myocardial tissues were measured by H&E staining. Normal mice exhibited a complete myocardial tissue structure with uniformly sized nuclei and normal myocardium space. There were no obvious pathological changes in myocardial tissues of normal rats. In CIH rats, myocardial tissues were arranged in a “wavy” shape, nucleus were different in size and arranged in disorder. Myocardial space was significantly enlarged, part of myocardium was ruptured, and some myocardial cells appeared degeneration and necrosis. These lesions of myocardial tissues were alleviated by *β-Sitosterol* at dose-dependent manner (Figure 1D). All these findings demonstrated that *β-Sitosterol* alleviated CIH-induced myocardial injury in rats at dose-dependent manner.

### 3.2 β-Sitosterol reduced the expression of MALAT1, HIF1A and apoptosis-related proteins in CIH rats

**FIGURE 2:**
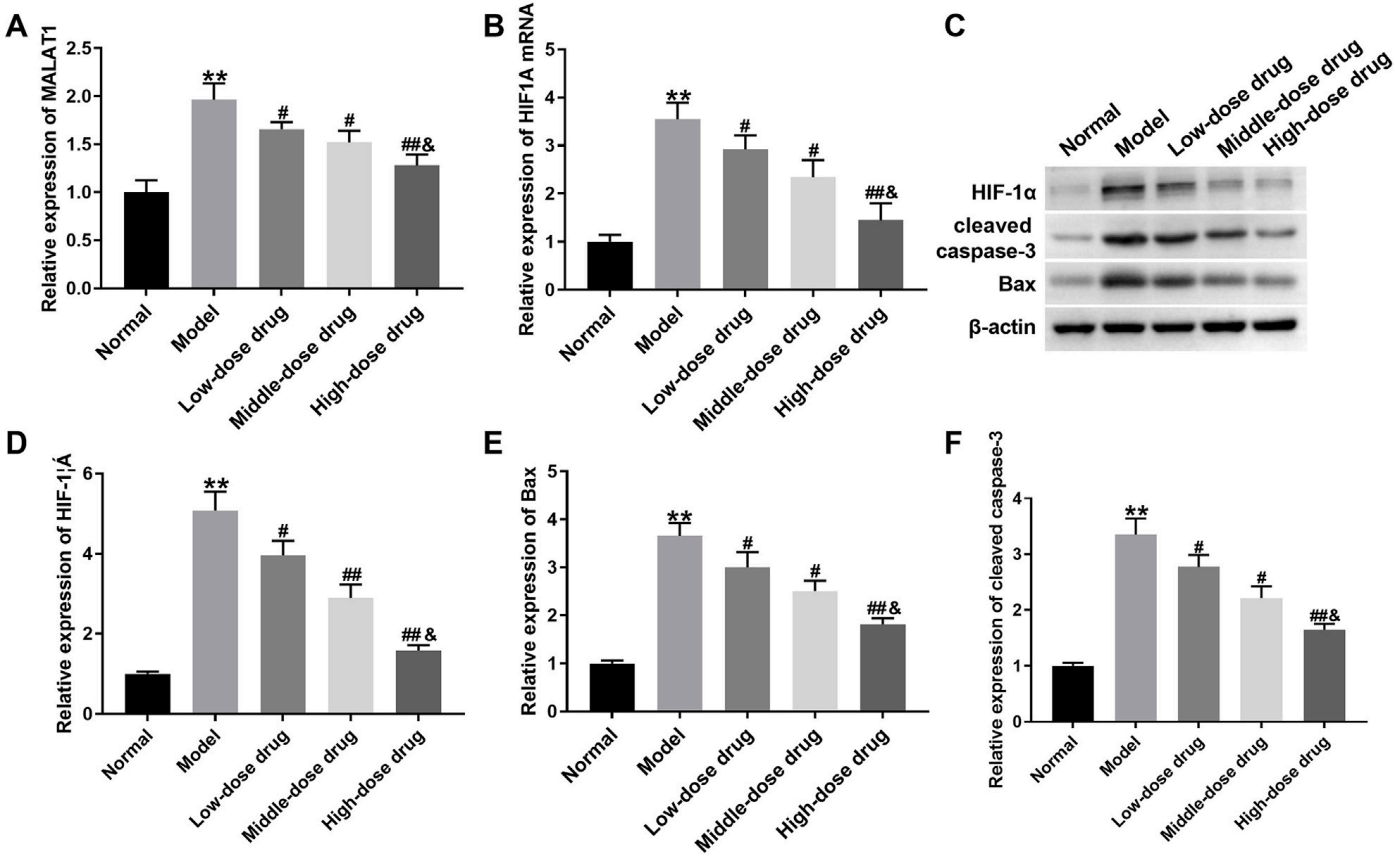
*β-Sitosterol* downregulates MALAT1/HIF1A and apoptosis-related proteins in CIH rats CIH rats were administrated with intermittent hypoxia, followed by low, middle and high dose of β-Sitosterol treatment. **(A,B)** qRT-PCR analysis of MALAT1 and HIF1A mRNA levels in myocardial tissues. **(C)** Representative Western blot bands and **(D–F)** quantitative analysis of HIF-1α, Bax, and cleaved caspase-3 protein levels (β-actin: loading control). Data are presented as mean ± SD; n = 8 per group. ^**^ *P* < 0.01 vs Normal; ^#^ *P* < 0.05, ^##^ *P* < 0.01 vs Model; ^&^ *P* < 0.01 vs Middle-dose drug.

To test whether *β-Sitosterol* regulated MALAT1 and HIF1A to alleviate myocardial injury in CIH rats, qRT-PCR detected the expression of MALAT1 and HIF1A in the myocardial tissues. MALAT1 and HIF1A were obviously elevated in myocardial tissues of CIH rats, which was suppressed by different dose of *β-Sitosterol*, especially high dose of *β-Sitosterol* (Figures 2A,B). Using Western blotting, we found that *β-Sitosterol* reduced CIH-mediated high expression of HIF-1α, Bax and cleaved caspase-3 in rats at dose-dependent manner (Figures 2C–F). Thus, these data suggest that *β-Sitosterol* reduced the expression of MALAT1, HIF1A and apoptosis-related proteins in CIH rats.

### 3.3 β-Sitosterol promoted viability and inhibited apoptosis of CIH-treated H9c2 cells at dose-dependent manner

**FIGURE 3:**
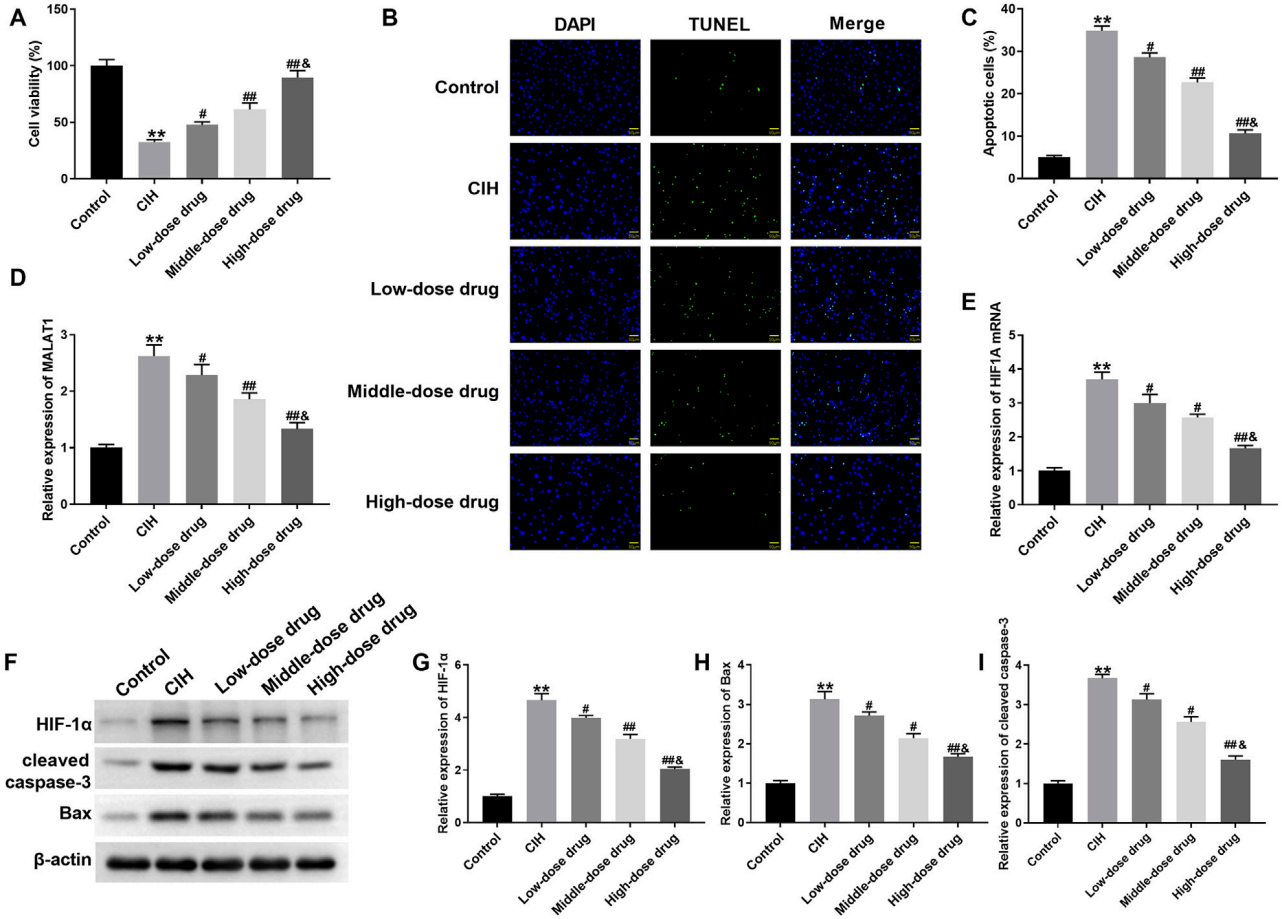
*β-Sitosterol* protects H9c2 cells against CIH-induced injury H9c2 cells were exposed to CIH environment, followed by low, middle and high dose of β-Sitosterol treatment. **(A)** Cell viability measured by CCK-8 assay. **(B)** Representative TUNEL staining (green: apoptotic cells; red: nuclei; scale bar: 20 μm) and **(C)** apoptosis rate quantification. **(D,E)** qRT-PCR analysis of MALAT1 and HIF1A mRNA. **(F)** Representative Western blot bands and **(G–I)** quantification of HIF-1α, Bax, and cleaved caspase-3. **P < 0.01 vs Control; #P < 0.05, ##P < 0.01 vs CIH; &P < 0.01 vs Middle-dose drug.

We further examined whether *β-Sitosterol* altered CIH-induced myocardial injury *in vitro*. Using CCK-8 assay, we found that cell viability of CIH-treated H9c2 cells was severely decreased, which was enhanced by different dose of *β-Sitosterol* at dose-dependent manner (Figure 3A). Results of TUNEL staining exhibited that *β-Sitosterol* significantly suppressed CIH-induced apoptosis of H9c2 cells, although at different extent (Figures 3B,C). Moreover, high levels of MALAT1 and HIF1A were observed in CIH-treated H9c2 cells. *β-Sitosterol* obviously reduced the levels of MALAT1 and HIF1A in CIH-treated H9c2 cells, especially high dose of *β-Sitosterol* (Figures 3D,E). The expression of HIF-1α, Bax and cleaved caspase-3 was notably increased in CIH-treated H9c2 cells. Different dose of *β-Sitosterol* led a downregulation of HIF-1α, Bax and cleaved caspase-3 in CIH-treated H9c2 cells at dose-dependent manner (Figures 3F–I). Therefore, *β-Sitosterol* promoted viability and inhibited apoptosis of CIH-treated H9c2 cells at dose-dependent manner.

### 3.4 MALAT1 could not bind to HIF1A and localized with HIF1A in the cytoplasm of CIH-treated H9c2 cells

**FIGURE 4:**
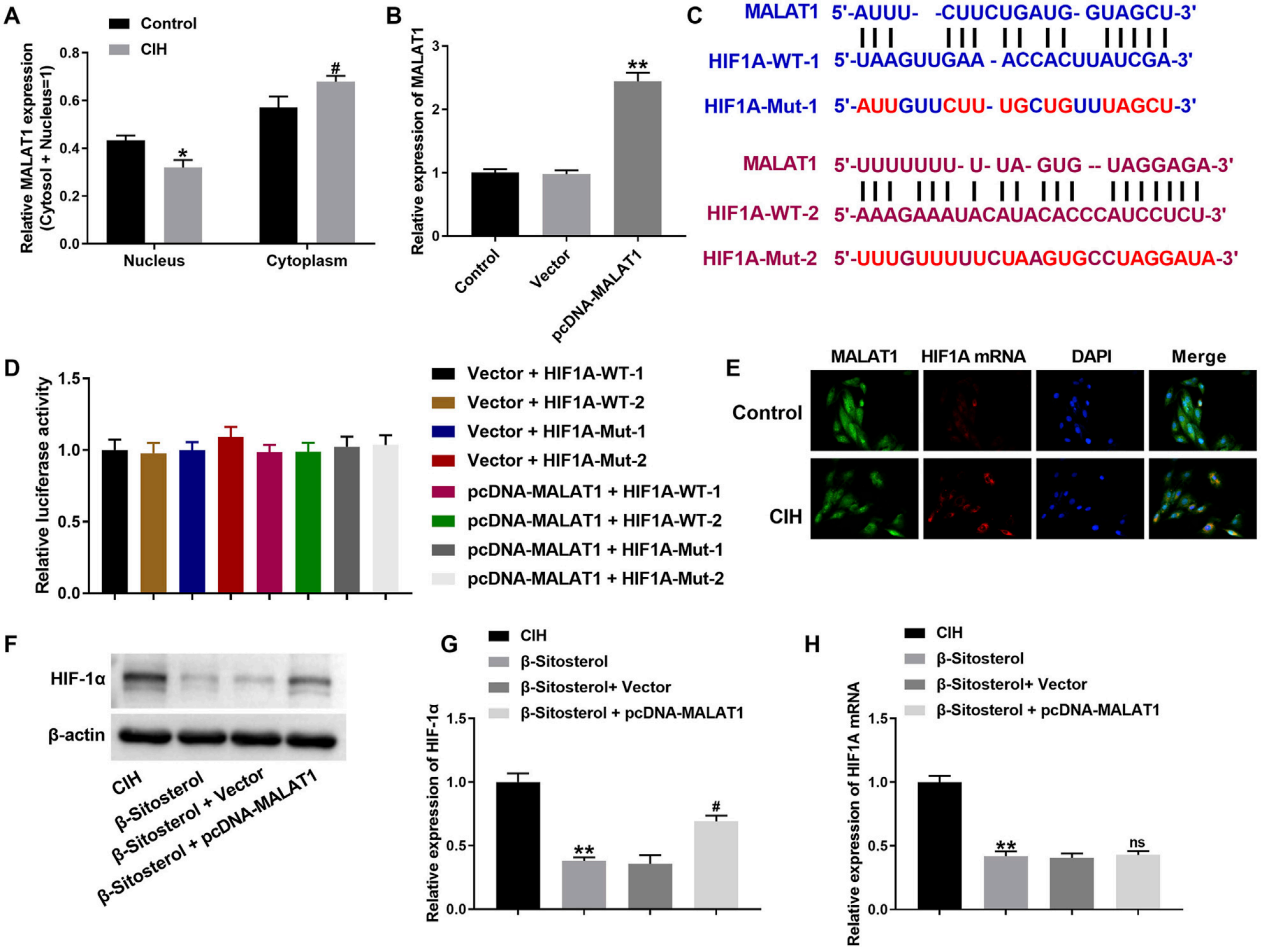
Cytoplasmic co-localization but no direct binding between MALAT1 and HIF1A. **(A)** Subcellular distribution of MALAT1 mRNA by qRT-PCR (Cyto: cytoplasm; Nuc: nucleus). **(B)** MALAT1 overexpression efficiency after pcDNA-MALAT1 transfection. **(C)** Predicted binding sites between MALAT1 and HIF1A (starBase database). **(D)** Luciferase reporter assay showing no interaction (ns: not significant). **(E)** RNA-FISH showing cytoplasmic co-localization of MALAT1 (green) and HIF1A (red) in CIH-treated cells (yellow: merged signals; scale bar: 10 μm). **(F–H)** qRT-PCR Western blot of HIF-1α/HIF1A in cells treated with β-sitosterol ± MALAT1 overexpression. **P < 0.01 vs CIH; #P < 0.01 vs β-Sitosterol + Vector; ns indicated P > 0.05.

We sought to verify the relationship between MALAT1 and HIF1A. Findings obtained from qRT-PCR uncovered that MALAT1 expression was decreased in nucleus, and increased in cytoplasm of CIH-treated H9c2 cells (Figure 4A). Then, we performed MALAT1 overexpression in H9c2 cells. MALAT1 was highly expressed in H9c2 cells in the presence of pcDNA-MALAT1 (Figure 4B). MALAT1 expression was increased approximately 3.5-fold in H9c2 cells transfected with pcDNA-MALAT1 compared to the vector control, which is consistent with pathologically relevant levels reported in hypoxia models (Luo et al., 2022). The starBase database predicated that MALAT1 may interact with HIF-1A. The potential binding sites between MALAT1 and HIF-1A were shown in Figure 4C. To verify the predication, luciferase reporter assay was carried out. The luciferase activity had no change in H9c2 cells following transfection of pcDNA-MALAT1 and WT/Mut of HIF-1A, suggesting that MALAT1 could not bind to HIF1A (Figure 4D). The results of RNA FISH revealed that MALAT1 translocated into the cytoplasm of CIH-treated H9c2 cells. Compared with normal H9c2 cells, the colocalization of MALAT1 with HIF1A was increased in CIH-treated H9c2 cells (Figure 4E). Moreover, *β-Sitosterol* caused a downregulation of HIF1A and HIF-1α in CIH-treated H9c2 cells. MALAT1 overexpression elevated HIF-1α expression, while had no influence on HIF1A expression in CIH-treated H9c2 cells in the presence of *β-Sitosterol* (Figures 4F–H). Thus, these results suggested that MALAT1 could not bind to HIF1A and localized with HIF1A in the cytoplasm of CIH-treated H9c2 cells.

### 3.5 β-Sitosterol promoted viability and inhibited apoptosis of CIH-treated H9c2 cells by regulating MALAT1 expression

**FIGURE 5:**
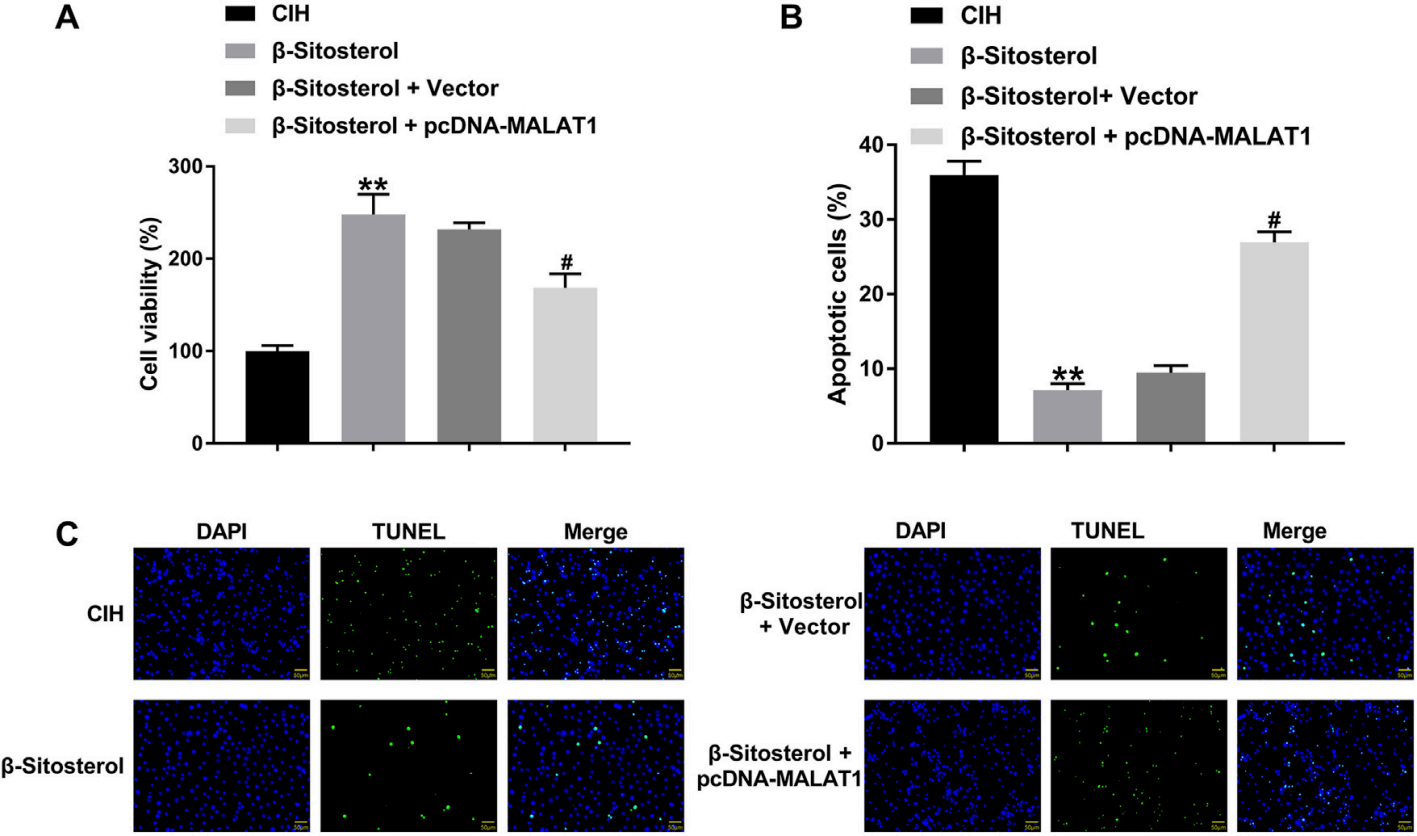
MALAT1 overexpression reverses *β-sitosterol*-mediated protection in H9c2 cells H9c2 cells were exposed to CIH environment, followed by β-Sitosterol treatment or/and MALAT1 overexpression. **(A)** Cell viability (CCK-8) and **(B,C)** apoptosis (TUNEL) in CIH-treated H9c2 cells with β-sitosterol ± MALAT1 overexpression. **P < 0.01 vs CIH; #P < 0.01 vs β-Sitosterol + Vector.

Using CCK-8 assay, we found that *β-Sitosterol* treatment elevated cell viability of CIH-treated H9c2 cells, which was abolished by MALAT1 overexpression (Figure 5A). TUNEL staining results uncovered that MALAT1 upregulation reversed *β-Sitosterol*-mediated inhibition of apoptosis in CIH-treated H9c2 cells (Figures 5B,C). Therefore, *β-Sitosterol* promoted viability and inhibited apoptosis of CIH-treated H9c2 cells by regulating MALAT1 expression.

### 3.6 β-Sitosterol alleviated CIH-induced myocardial injury in rats by regulating MALAT1 expression

**FIGURE 6:**
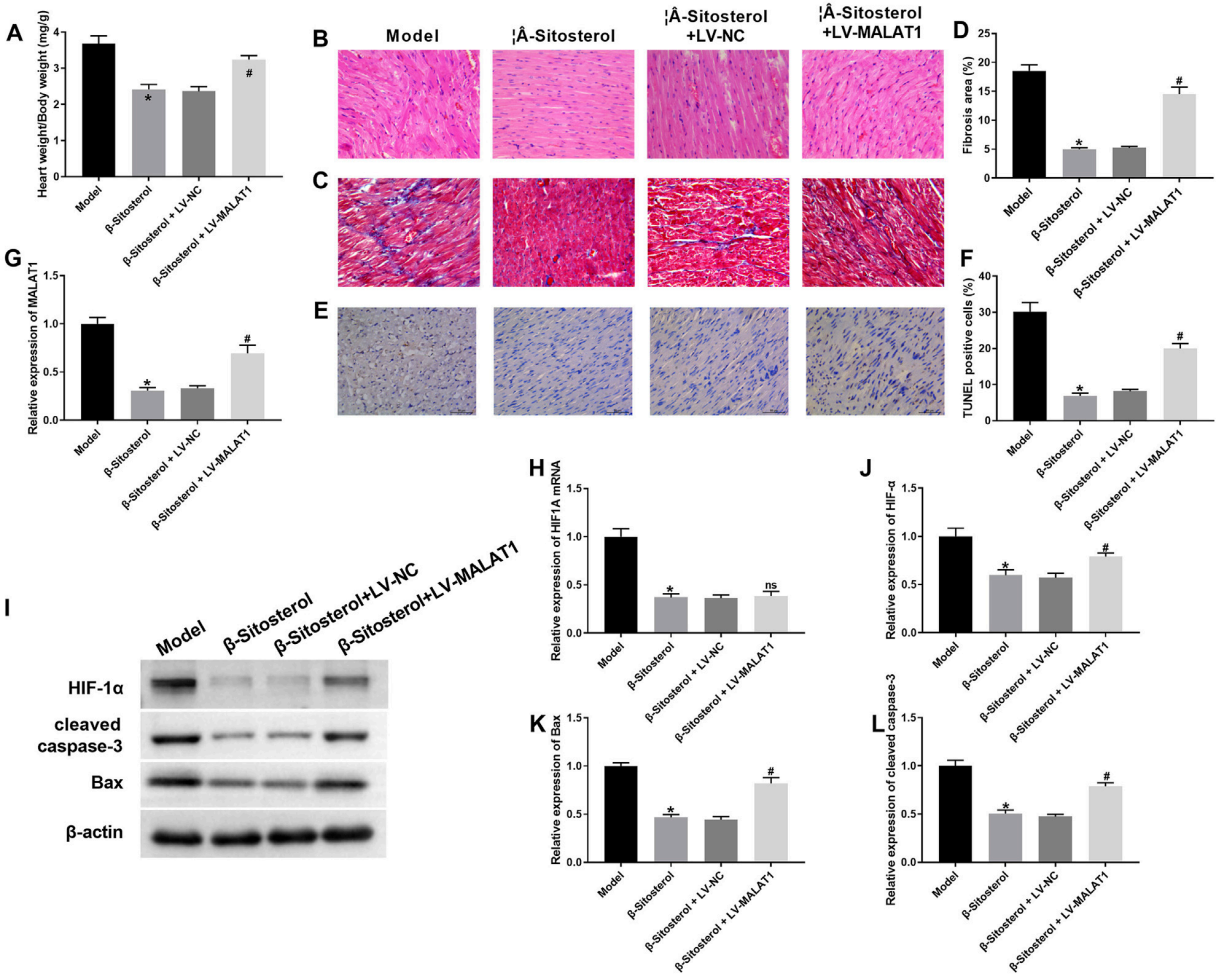
*β-Sitosterol* alleviates myocardial injury via MALAT1 *in vivo*. CIH rats were induced by administration of intermittent hypoxia, followed by *β-Sitosterol* treatment or/and MALAT1 overexpression. **(A)** HW/BW ratio. **(B)** Representative H&E staining of myocardial pathology (scale bar: 50 μm). **(C,D)** Masson’s staining (fibrosis, blue) and **(D)** quantification. **(E)** TUNEL staining (apoptosis, green) and **(F)** quantification. **(G,H)** qRT-PCR of HIF1A mRNA. **(I)** Western blot bands and **(J–L)** quantification of HIF-1α, Bax, and cleaved caspase-3. Data are presented as mean ± SD; n = 8 per group. ^*^ *P* < 0.05 vs Model; ^#^ *P* < 0.05 vs β-Sitosterol + LV-NC; ns indicated *P* > 0.05.

Finally, we explored whether *β-Sitosterol* alleviated CIH-induced myocardial injury by regulating MALAT1 *in vivo*. *β-Sitosterol*-mediated decreased heart weight/body weight in CIH rats was abrogated by MALAT1 overexpression (Figure 6A). Using H&E staining, we found that *β-Sitosterol* effectively alleviated the pathological damages of myocardial tissues in CIH rats. The influence conferred by *β-Sitosterol* was abolished by MALAT1 overexpression (Figure 6B). Additionally, the fibrosis and apoptosis of myocardial tissues was examined by Masson and TUNEL staining. As shown in Figures 6C–F, MALAT1 overexpression reversed *β-Sitosterol*-induced inhibition of fibrosis and apoptosis of myocardial tissues in CIH rats. The expression of HIF1A, HIF-1α, Bax and cleaved caspase-3 was decreased in CIH rats following *β-Sitosterol* treatment. MALAT1 upregulation had no influence on HIF1A expression, significantly enhanced the expression of HIF-1α, Bax and cleaved caspase-3 in *β-Sitosterol*-treated CIH rats (Figures 6H–L). Thus, *β-Sitosterol* alleviated CIH-induced myocardial injury in rats by regulating MALAT1 expression.

## 4 Discussion

As a global sleep disorder problem, OSA adds to the burden on public health. In this work, we constructed a CIH rat model to investigate the influence of *β-Sitosterol* on OSA *in vivo*. We found that *β-Sitosterol* effectively reduced heart weight/body weight, alleviated myocardial fibrosis, apoptosis and injury in CIH rats at dose-dependent manner. The expression of MALAT1 and HIF1A was significantly increased in CIH rats, which was repressed by *β-Sitosterol* treatment. *In vitro*, *β-Sitosterol* promoted cell viability and inhibited apoptosis of CIH-treated H9c2 cells at dose-dependent manner. In terms of mechanism, MALAT1 could not interact with HIF1A, MALAT1 and HIF1A co-localized in the cytoplasm of CIH-treated H9c2 cells. *β-Sitosterol* alleviated CIH-induced myocardial injury *in vivo* and *in vitro* by down-regulating MALAT1 and HIF1A.

*β-Sitostero*l is one of the phytosterols with various physiological activity and high nutritional value (Khan et al., 2022). It is widely used in medicine, healthcare products, cosmetics and other fields. For instance, Chen et al. have demonstrated that *β-sitosterol* reduces cell apoptosis by limiting the expression of apoptosis-related genes, Bax, Caspase-9 and Caspase-3, thereby exerting protective effect on alcohol-induced liver damage (Chen et al., 2020). As an active ingredient of *Panax Notoginseng* (Burk.), *β-sitosterol* may has a vital role in the inhibitory effect of *Panax Notoginseng* on myocardial fibrosis (Han et al., 2022). A previous study has found that *β-sitosterol* inhibits the levels of inflammatory response and oxidative stress, which contributes to alleviate apoptosis and necrosis of renal and cardiac tissues (Koc et al., 2021). Consistently, we also confirmed the cardioprotective effect of *β-sitosterol* on myocardial injury. *β-Sitosterol* alleviated CIH-induced myocardial injury in rats at dose-dependent manner. *In vitro*, *β-Sitosterol* promoted cell viability and reduced apoptosis of CIH-treated H9c2 cells at dose-dependent manner. *β-Sitosterol* treatment repressed the expression of pro-apoptotic proteins, Bax and cleaved caspase-3, in CIH rats and CIH-treated H9c2 cells. The activated caspase-3, cleaved caspase-3, is the only way for cell apoptosis, which indicates the beginning of cell apoptosis program and is the sign of apoptosis entering an irreversible stage (Brentnall et al., 2013). Thus, *β-Sitosterol* exerted cardioprotective effect on CIH-induced myocardial injury at dose-dependent manner. While HW/BW ratio and histology are well-established indicators of myocardial remodeling (Sun et al., 2023), future studies incorporating echocardiography or molecular markers like collagen and TGF-β1 would provide further functional insights.

MALAT1 is a well-studied lncRNA, which is involved in the occurrence and development of various diseases, such as cancer, cerebral ischemic stroke, diabetic foot ulcer, etc. (Goyal et al., 2021; Wang et al., 2020; Jayasuriya et al., 2020). Additionally, MALAT1 silencing exerts cardioprotective effects on sepsis-induced myocardial injury by reducing inflammation and apoptosis of cardiomyocytes through miR-26a-5p/Rcan2 axis (Luo et al., 2022). Liu et al. utilized hypoxia/reoxygenation-treated H9c2 cells and ischemia reperfusion rat model to test the functional role of MALAT1 in myocardial ischemia-reperfusion injury (Liu et al., 2022). MALAT1 knockdown regulates miR-133a-3p/IGF1R axis to alleviate hypoxia/reoxygenation-induced myocardial apoptosis and damage *in vivo* and *in vitro*. In this work, we investigated the biological role of MALAT1 in CIH-induced myocardial injury. Upregulation of MALAT1 was observed in CIH-treated rats and H9c2 cells. MALAT1 overexpression enhanced cell apoptosis and aggravated CIH-induced myocardial injury. Although MALAT1 knockdown was not performed in this study, our overexpression data strongly support the role of MALAT1 in mediating CIH-induced injury. Previous studies have shown that MALAT1 silencing attenuates myocardial apoptosis (Luo et al., 2022; Liu et al., 2022), consistent with our findings. Mechanism studies showed that MALAT1 did not bind to HIF1A and localized with HIF1A in the cytoplasm of CIH-treated H9c2 cells. MALAT1 overexpression enhanced the expression of HIF-1α, and had no influence on HIF1A expression in H9c2 cells. Although MALAT1 co-localized with HIF1A in CIH-treated H9c2 cells, it did not directly affect its function. Although no direct binding was observed, MALAT1 may act as a scaffold for RNA-binding proteins (e.g., HuR or AUF1) that stabilize HIF1A mRNA or enhance its translation under hypoxia, ultimately elevating HIF-1α protein levels. *β-Sitosterol* likely disrupts this complex formation. Bioinformatics analysis via starBase predicted potential interactions between MALAT1 and RNA-binding proteins involved in mRNA stability (e.g., HuR). Future studies should explore whether MALAT1 recruits such proteins to regulate HIF1A post-transcriptionally. Further studies are needed to identify the specific proteins involved. MALAT1 may promote the expression of HIF-1α by recruiting other proteins to HIF1A mRNA, which needs further exploration.

In summary, this work demonstrated that *β-Sitosterol* ameliorated chronic intermittent hypoxia-induced myocardial injury by down-regulating MALAT1 and HIF-1A. Thus, *β-Sitosterol* may be a potential drug for OSA treatment. As a natural compound with low toxicity, *β-sitosterol* could serve as an adjunctive therapy for OSA patients intolerant to CPAP, potentially reducing cardiovascular complications.

## Data Availability

The original contributions presented in the study are included in the article/supplementary material, further inquiries can be directed to the corresponding author.

## Abbreviations

CIH, Chronic intermittent hypoxia; CPAP, Continuous positive airway pressure; HIF-1α, Hypoxia-inducible factor-1α; LV, Lentivirus; LV-NC, LV-mediated empty vector; lncRNAs, Long non-coding RNAs; OSA, Obstructive sleep apnea; qRT-PCR, Quantitative real-time PCR; SD, Sprague Dawley.
